# Prodrome or risk syndrome: what’s in a name?

**DOI:** 10.1186/s40345-017-0077-5

**Published:** 2017-04-10

**Authors:** Pierre Alexis Geoffroy, Jan Scott

**Affiliations:** 1 U1144, Inserm, 75006 Paris, France; 2 Pôle de Psychiatrie et de Médecine Addictologique, AP-HP, GH Saint-Louis – Lariboisière – F. Widal, 75475 Paris, France; 3 Academic Psychiatry, Institute of Neuroscience, Wolfson Unit, Newcastle University, Newcastle upon Tyne, UK

**Keywords:** Prodrome, Risk syndrome, Bipolar disorder, Bipolar at risk, Psychosis, Ultra-high risk

## Abstract

**Background:**

In the last decade, an increasing number of publications have examined the precursors of bipolar disorders (BD) and attempted to clarify the early origins and illness trajectory. This is a complex task as the evolution of BD often shows greater heterogeneity than psychosis, and the first onset episode of BD may be dominated by depressive or manic features or both. To date, most of the published reviews have not clarified whether they are focused on prodromes, risk syndromes or addressing both phenomena. To assist in the interpretation of the findings from previous reviews and independent studies, this paper examines two concepts deemed critical to understanding the pre-onset phase of any mental disorder: prodromes and risk syndromes. The utility of these concepts to studies of the evolution of bipolar disorder (BD) is explored.

**Findings:**

The term “prodrome” is commonly used to describe the symptoms and signs that precede episode onset. If strictly defined, the term should only be applied retrospectively as it refers to cohorts of cases that all progress to meet diagnostic criteria for a specific disorder and gives insights into equifinality. Whilst prodromes may reliably predict individual relapses, the findings cannot necessarily be extrapolated to identify prospectively who will develop a first episode of a specific disorder from within a given population. In contrast, ‘risk syndrome’ is a term that encompasses sub-threshold symptom clusters, but has often been extended to include other putative risk factors such as family history, or other variables expressed continuously in the population, such as personality traits. Only a minority of individuals ‘at risk’ make the transition to a specific mental disorder. By prospectively observing those cases where the risk syndrome does not progress to severe disorder or progress to a non-BD condition, we gain insights into the discriminant validity of different pre-BD characteristics, pluripotentiality of outcomes, and protective factors and resilience.

**Conclusion:**

We emphasize the clinical and research utility of prodromes and risk syndromes, examine examples of the conflation of the concepts, and highlight the rationale for regarding them as discrete entities.

## Background

Mental health services around the world are increasingly adopting a policy of early intervention (EI) for young adults with severe mental disorders. This began with a focus on psychosis—initially promoting early secondary intervention for new onset cases, with two main goals: timely treatment and followed by extended support throughout the ‘critical period’ (about 3–5 years after first episode onset) (McGorry et al. 2010; Norman et al. 2011; Srihari et al. 2012). Latterly, a number of services have tried to identify individuals who are at ‘ultra-high risk’ (UHR) of developing psychosis, but whose symptoms are sub-threshold for current diagnostic criteria (Yung et al. 2005). The latter has fostered the development of standardized assessment instruments that identify those help-seeking individuals with subjective distress and impaired functioning who can be categorized as UHR cases (e.g. those with schizotypal personality and family history of psychosis, attenuated psychotic symptoms, or brief intermittent psychotic symptoms, etc.) (Yung et al. 2005). It is proposed that identifying those at highest risk offers a unique opportunity to delay or prevent transition from UHR to first episode of psychosis, and current evidence indicates that interventions can reduce predicted transition rates by about 50% over about 2 years (Marshall and Rathbone 2011).

Unsurprisingly, there is now considerable interest in exploring if these concepts and strategies can be translated from psychosis to other severe mental disorders, especially bipolar disorders (BD), which also have a peak age of onset in late adolescence and early adulthood (Geoffroy et al. 2013a; Jones 2013; Merikangas et al. 2012). To improve the prospects for EI in BD, clinicians need to be able to identify individuals with the earliest manifestations of a sub-threshold presentation and/or other risk markers of BD, and to be able to predict transitions, e.g. to a first manic episode, even in cases where the course of illness may show discontinuities (e.g. cases where a first depressive episode is followed by euthymia and/or further depressions, then later by hypomanic symptoms, etc.).

In contrast to psychosis, a problem for research in BD is that there are fewer prospective studies of early transition from being ‘at risk of BD’ to syndromal disorder (Faedda et al. 2014; Malhi et al. 2014; Scott et al. 2016). So far, the clinical cohort studies undertaken in general psychiatry or early intervention services have mainly recruited BD cases that already fulfil diagnostic criteria for an episode, and so the evolution and characteristics of the sub-threshold manifestations are reconstructed retrospectively (Correll et al. 2007, 2014). Alternatively, enrichment strategies have been used, such as recruiting the offspring of BD parents (e.g. Duffy et al. 2011). Whilst useful, these studies cannot always help clinicians in day-to-day practice, as most youth presenting with BD seen in general settings do not have a parent or other close family member with BD (National Institute of Healthcare and Clinical Excellence; NICE 2014). Indeed, the recent NICE guideline on BD suggests that the presence of family history of BD in cases of depression should not be used to identify potential risk of BD (NICE 2014) as it predicts both recurrent unipolar depression (UP) as well as BD and genetic loading for BD alone may not be sufficiently discriminatory (NICE 2015). Whilst this advice is important, we suggest that clinicians and researchers will still gain important insights from interviewing family members on a face-to-face basis to help in assessing the meaning of behavioural problems and diagnoses in children and adolescents who meet ‘bipolar at risk’ (BAR) criteria (including offspring of parents with BD), and in determining putative illness trajectories and paradoxical responses to treatment (Grof et al. 2009).

Although a number of reviews have been published that summarize the evidence regarding putative risk factors or syndromes for BD, these frequently intermingle the findings from heterogeneous combinations of retrospective studies of prodromes and prospective studies of risk syndromes or other BAR categories (Howes et al. 2011; Bechdolf et al. 2012; Brietzke et al. 2012; Leopold et al. 2012). In this paper, we propose that whilst both types of information may be useful in understanding elements of the evolution of BD, conflating findings regarding BAR syndromes with those from ‘prodrome’ studies can be a source of confusion about the critical variables and symptoms that can accurately predict transition to BD. We offer a rationale for discriminating between the two constructs, and illustrate why it is important that they are considered separately in the future.

## Defining the concepts

### Prodromes

The word “prodrome” means ‘a forerunner of an event’. In psychiatry, Fava and Kellner (1991) have defined it as follows:

the early symptoms and signs that precede the acute clinical phase of an illness.

Prodromal symptoms or signs may precede the full episode syndrome by weeks or months. However, if the term is accurately applied, a prodrome is always a precursor of illness onset, and it indicates that the initial symptoms developed into an episode of disorder meeting defined criteria (Eaton et al. 1995; Simon et al. 2001), i.e. a prodrome can only be fully described after disease onset.

A source of confusion has been that some researchers have failed to consider that prodromal symptoms should be continuous with the acute illness phase of the disorder. In the past, the term ‘early prodrome’ has been (erroneously) applied to childhood antecedents, such as pre-pubertal anxiety syndromes. However, these are neither a systematic prodrome of adult mental disorder, nor are they specific to BD (Rubino et al. 2009; Scott et al. 2013; Skjelstad et al. 2010). The concept of the early prodrome is better understood as a point of ‘no return’, i.e. the ‘early prodrome’ represents an irreversible process that progresses into the ‘late prodrome’ (Eaton et al. 1995; Faedda et al. 2015), which in turn evolves into an illness episode (unless treatment interventions interrupt the process). These childhood clinical phenotypes in individuals who later develop BD are not an ‘early prodrome’ as they are rarely continuous with the adult phenotypes, but represent risk markers or the very early stage of the BD (or possibly other disorders) (Eaton et al. 1995; Kim-Cohen et al. 2003).

Prodrome studies mainly focus on relapses in established BD, and are rarely limited to the signs and symptoms preceding the first episode alone (Correll et al. 2007; Jackson et al. 2003). In many but not all studies, the description of early warning signs of relapse may include reference to potential triggering events that immediately precede symptom onset or that are closely associated with symptom escalation (Lobban et al. 2010; Morriss et al. 2007). Clinically, defining the boundary between the late prodrome and the actual onset of an episode can be difficult. Indeed, distinguishing the point when a person meets criteria for a relapse of BD depression is complicated as many individuals have persistent sub-syndromal inter-episode symptoms (Judd et al. 2002), rather than discrete episodes (Jackson et al. 2003; Morriss et al. 2007). The term prodrome has also been used as a narrow (e.g. the prodrome for a manic episode) or a broader concept (e.g. studies of depressive prodromes that include BD and unipolar cases), and/or as reported in some studies of prodromes for psychosis that do not distinguish between affective or schizophreniform psychoses (Bechdolf et al. 2010; Correll et al. 2007; Fusar-Poli et al. 2013; Jackson et al. 2003; Thompson et al. 2011). Studies of prodromes offer insights into the equifinality of different prodromal signs and symptoms.

Prodromes can have individual validity, as more than half of BD cases can reliably identify three or more features that consistently indicate the early stages of a manic or depressive relapse (Jackson et al. 2003). For established cases, there is often sufficient intra-individual consistency in the temporal sequence of the symptoms to allow the initiation of personalized interventions to avert a potential relapse (Jackson et al. 2003; Morriss et al. 2007; Perry et al. 1999; Scott 2011). At a population level, prodromes can also have group validity in observing general patterns of the pathophysiology such as slow-onset mania prodromes with slow or rapid deterioration, or rapid-onset-and-deterioration prodrome (Correll et al. 2014). Studies of prodromes may also make it feasible to identify biomarkers of BD relapse and whether these differ between depression and (hypo)mania (Kapczinski et al. 2009).

### Risk syndromes

According to Garmezy (1983), and Werner and Smith (1992), risk factors can be defined as follows:

those characteristics, variables, or hazards that, if present for a given individual, make it more likely that this individual, rather than someone selected from the general population, will develop a disorder.

Risk can be expressed continuously in a population (e.g. the distribution of certain personality traits), but in high-risk research, phrases such as ‘UHR’ or ‘BAR’ criteria are often used categorically. The phrase ‘risk syndrome’ can be used to describe this amalgamation of one or more clinical symptoms and/or other putative precursors that together may increase the likelihood of transition to BD, such as a combination of state, trait and familial markers (Yung et al. 2005; Bechdolf et al. 2010; Bechdolf et al. 2014; Scott et al. 2016). Whilst risk factors may be discussed in the context of both first and recurrent episodes, the literature usually specifies if the study is about risk of recurrence and most research on BAR (or UHR for psychosis) restricts the use of the term ‘risk syndrome’ to the exploration of the presence or absence of a specified combination of antecedents in relation to first episode onset (Scott 2012).

Studies of risk offer the opportunity for prospective comparison of individuals who do or do not develop BD within a specified time period. Currently, the critical limitation to this research is that the sample sizes are often small and the operationalization of the putative risk syndrome for BD is not standardized; even when the same criteria are employed across studies, the assessment tools may vary (Correll et al. 2007; Bechdolf et al. 2010). Also, there is the potential for confounding in some evaluations such as quantitative measures of trait personality such as cyclothymia (as these features may overlap with symptoms of BD) (Scott 2012; Scott et al. 2016). As the outcome of the participants is unknown at initial at assessment, the impact of these problems theoretically applies equally across the entire cohort, but it is a potential bias that is yet to be eliminated. As a consequence, prospective studies of risk syndromes allow researchers to measure and observe multiple outcomes, e.g. homotypic or heterotypic continuity of BAR, or a return to health (Geoffroy et al. 2013b), and give insights into multifinality. In a recent systematic review (Faedda et al. 2014), 16 prospective studies were examined to explore clinical risk factors for BD onset. Although some consistencies in pre-onset features were reported, there was no consideration of other (non-BD) outcomes, so the sensitivity, specificity, predictive values and clinical utility of these clinical risk factors were not estimated.

It is possible to undertake a study of risk syndromes using the ‘follow-back’ methodology (Kim-Cohen et al. 2003). For example, Bechdolf et al. (2010) reported an audit of the initial structured clinical assessments undertaken with individuals attending youth mental health services and identified those who did or did not meet pre-defined BAR criteria (sub-threshold manic symptoms; or history of depression plus cyclothymia or a family history of BD). The transition rate to BD was 22% over 12 months in those deemed at risk, compared to 0.7% in individuals who did not meet the criteria. Scott (2012) reported similar levels of sensitivity (0.86) and specificity (0.72) using these criteria in a follow-back study using a ‘case:positive control’ design (BD vs. other mental disorders), and recently, Scott et al. (2016) examined which clinical characteristics showed optimum utility for identifying which depressed youth made an early transition to BD (within 2 years). Cyclothymia showed the best discriminant validity for case finding and screening out those who would not make transition, with sub-threshold manic symptoms being ranked as the second most useful factor. Family history of BD, atypical depressive symptoms and antidepressant-induced elation were useful for screening.

For clinicians, prospective studies of risk criteria have the advantage of closely reflecting the reality of day-to-day practice. As only about 20–30% of individuals who meet a set of pre-defined BAR criteria will develop a BD syndrome meeting diagnostic criteria (Bechdolf et al. 2010), and the clinician has to apply research evidence to estimate the likelihood of transition to clinical caseness and then plan any interventions accordingly (Axelson et al. 2011). Early identification of those at above-average risk can be offered prospective monitoring that allows for more intensive early intervention if symptoms escalate. A recent meta-analysis observed that the initial prodromal period, whilst quite extended (about 27 months), is characterized by symptoms that are largely consistent with the subsequent mood episode (Van Meter et al. 2016). Of course, a further potential benefit of studying risk syndromes is that it offers a means to link clinical phenotypes to endophenotypes and biomarkers to examine their validity (Hickie et al. 2013).

## Differentiating prodromes and risk syndromes

A source of confusion for many researchers is that precursor syndromes or putative risk factors reported in retrospective studies of prodromes and prospective studies of risk syndromes may overlap. A classic example is the high frequency of anxiety disorders reported in individuals who develop BD. In retrospective studies of individuals with an established diagnosis of BD, up to 75% of cases may report anxiety as a feature of the prodromal phase. However, this finding does not mean that prospectively, 75% of individuals with anxiety will develop BD (under a given set of circumstances). Prospective follow-ups demonstrate that, not only is anxiety a non-specific risk factor for BD (being part of the developmental trajectory of a range of mental disorders), but also that it actually occurs more frequently prior to the first onset of UP and of psychosis (in many studies) (e.g. Kim-Cohen et al. 2003; Rubino et al. 2009). So, although a symptom might be an important feature of prodrome (e.g. increasing risk of relapse), and have high prevalence in those with the diagnosis, its utility as a core feature of BAR that prospectively predicts onset of the disorder is largely determined by its specificity.

The key difference between a ‘prodrome’ and ‘risk syndrome’ is that the former is primarily a predictor of the onset of an episode of the mental disorder under examination, whilst the latter is primarily a predictor of the overall likelihood that someone will experience a *first* onset of a disorder (compared to no disorder or another disorder). In Table 1, we highlight the main characteristics of studies of prodromes compared to risk syndromes.

**Table 1:** Main characteristics of studies of prodromes and risk syndromes

	Prodrome studies	Risk studies
Primary focus	Symptoms and signs preceding any episode onset for a specified disorder	Predictive validity of ‘risk markers’ for 1st episode onset (versus no disorder or versus a different disorder)
Design	Retrospective	Prospective
Outcome	New episode of a specified disorder	Several possible outcomes (i.e. agnostic): presence or absence of disorder(s)
Reliability (+ positive; − negative)	+ High for late prodrome − Boundary between final phase of a prodrome difficult to differentiate from episode onset	+ High levels for most features − Less for some factors e.g. reporting of family history of mental disorders affected by lack of information or recall biases e.g. potential confounding of trait measures with prodromal symptoms
Statistical analysis	Within group comparisons only (100% of the sample = Cases)	Between group analyses: Cases vs. Controls
Limitations	Sampling biases Recall and response biases Lack of generalizability to studies of risk markers	Operationalization of risk syndromes and methods for measuring any specific criteria are not standardized In bipolar disorders, there is a lack of consensus regarding the goal, i.e. risk of onset of mania or of hypomania and mania (or of mixed states or affective psychosis) High resource use and costly (due to e.g. sample size requirements and/or duration of follow-up)
Advantages	Benefits for individuals can be instigated in the short-term (e.g. introduction of individualized relapse prevention programmes focused on early warning signs and symptoms) Inexpensive; relatively easy to plan and undertake	Can estimate likelihood of onset of a range of disorders in individuals and populations that have similar or different levels of risk Opportunities for use of enriched strategies (e.g. offspring studies) Identification of putative protective factors and/or exploration of interactions between risk and protective factors will inform prevention and early intervention strategies

For BD, the critical aspects of interpreting findings on prodromes are as follows:

- By definition, the key features of a prodrome are identified retrospectively after the symptoms experienced by the individual have progressed to meet the threshold criteria for episode onset (i.e. the person is by definition a ‘case’). The corollary is that if the symptoms do not meet diagnostic ‘caseness’, the individual cannot be described as experiencing a prodrome associated with the specified condition. An important implication for future studies of BD prodromes is that researchers need to decide a priori if they regard hypomanic and manic prodromes as part of a continuum or as separate disorders.
- It is inappropriate to combine retrospective findings regarding the nature or prevalence of symptoms of a BD prodrome with data from prospective studies of ‘at risk’ populations in which only a small proportion of individuals will become first episode cases. Data from the former are not generalizable as they derive from a within group analysis of the prodrome in a population comprising only of cases.

The critical aspects of studies of risk syndromes for BD are as follows:

- The study outcome is the proportion of participants putatively at risk of developing BD who made the transition to BD caseness versus those who did not; this means that conclusions about risk variables are based on between group analyses, within a specified time period.
- As only a proportion of individuals in the ‘at risk’ group will make the transition to clinical caseness, it allows assessment of the sensitivity, specificity, predictive validity and clinical utility of specific risk factors or symptom clusters for BD onset.
- Individuals deemed at risk of BD who do not develop the disorder need to be assessed carefully, as this sub-group can provide important insights into population protective factors or individual resilience; also, lack of transition to BD, does not mean the individual is free of disorder (e.g. they may have recurrent depression, etc.).

## Conclusions

Whilst prodromes may have less research utility for understanding first episode onset in mental disorders, they are particularly important in developing clinical strategies to prevent relapse (by recognizing and managing the relapse signature). Research in the field may benefit from explicitly adopting an approach (that is common in psychosis), of differentiating between the initial prodrome reported by individuals who meet criteria for a specific diagnosis, from prodromes for recurrence, especially as the latter is an important treatment target.

Research into BAR features is in its infancy and has yet to overcome several problems that undermine reliability and validity (e.g. no uniformity in the operationalization of risk syndrome criteria; reliance on small or heterogeneous samples; selection of and reliability of assessments varies; etc.). Furthermore, there is a lack of consensus in the literature on the ‘outcome condition’ selected, e.g. some studies choose an end-point of bipolar spectrum disorder, whilst other restrict the assessment of risk factors to the study of mania (and may include spectrum disorders as risk factors for this outcome). Given the range and diversity of presentations of the spectrum disorders and evidence that they may be precursors of BD-I (Axelson et al. 2011), we suggest that research on BAR syndromes should initially focus on transitions to mania, as it has higher reliability and greater clinical validity (Freedman et al. 2013; Hickie et al. 2013; Scott et al. 2013). Also, studies of prodromes and risk syndromes might benefit from the application of non-linear dynamic statistical analyses of real-time recordings of longitudinal data to allow a more nuanced approach to understanding the evolution of these phenomena (e.g. Glenn et al. 2006; Moore et al. 2014).

Lastly, even if causal risk factors are identified, the potential benefits of this research will only be realized if it is followed by attempts to establish (a) which components of the risk syndrome are modifiable, and (b) how these can be altered through interventions (Eaton et al. 1995; Mrazek and Haggerty 1994). As noted by Mrazek and Haggerty (1994), one of the major advantages of this risk syndrome approach is that it also emphasizes the interplay between risk and protective factors for the study population. This will be a critical scientific step in understanding transition from risk syndrome to full-blown disorder, and will also inform the strategy for planning and implementation of successful programmes of early intervention for individuals with emerging BD.

## Abbreviations

BD: bipolar disorders
BAR: bipolar at risk
UHR: ultra-high risk
